# Examining Youth Dual and Polytobacco Use with E-Cigarettes

**DOI:** 10.3390/ijerph15040699

**Published:** 2018-04-08

**Authors:** Youn Ok Lee, Jessica K. Pepper, Anna J. MacMonegle, James M. Nonnemaker, Jennifer C. Duke, Lauren Porter

**Affiliations:** 1 RTI International, Public Health Research Division, Research Triangle Park, NC 27709, USA; jpepper@rti.org (J.K.P.); amacmonegle@rti.org (A.J.M.); jnonnemaker@rti.org (J.M.N.); jduke@rti.org (J.C.D.); 2 Florida Department of Health, 4052 Bald Cypress Way, Tallahassee, FL 32399, USA; lauren.porter@flhealth.gov

**Keywords:** electronic cigarettes, cigarettes, other tobacco products, polytobacco use, psychosocial factors

## Abstract

E-cigarettes and other non-cigarette tobacco products are increasingly popular among youth. Little is known to inform public health efforts to reduce youth use. We examined psychosocial correlates of single and multiple tobacco product use among youth e-cigarette users. Data were from the 2014 Florida Youth Tobacco Survey (*N* = 69,923), a representative sample of Florida middle and high school students. Associations between combinations of e-cigarette, cigarette and other tobacco product (OTP) use and psychosocial variables were examined using multinomial logistic regression with an analytic sample of *N* = 2756. Most e-cigarette-using youth used at least one other product (81%). Perceiving cigarettes as easy to quit was significantly associated with greater likelihood of combined e-cigarette/OTP use (relative risk ratio (RRR) = 2.51, *p* < 0.001) and combined e-cigarette/cigarette/OTP use (RRR = 3.20, *p* < 0.0001). Thinking you will be smoking cigarettes in 5 years was associated with product combinations that include cigarettes. Tobacco company marketing receptivity was associated with multiple product user types. Given that specific psychosocial factors put youth at risk for concurrent use of e-cigarettes with tobacco products, public health efforts should address polytobacco use specifically, instead of individual product use. Youth perceptions about the ease of quitting cigarettes, intentions to continue smoking cigarettes and receptivity to tobacco company marketing are promising areas for messaging aimed at reducing e-cigarette polytobacco product use.

## 1. Introduction

With the introduction of electronic cigarettes (e-cigarettes) and their subsequent diversification into hundreds of brands and models [1] the marketplace of tobacco products in the United States is becoming increasingly complex and patterns of use are evolving. As rates of combustible cigarette use among U.S. youth decline, rates of hookah and e-cigarette use are increasing [2,3,4,5].

Many e-cigarette-using youths are dual or poly-users (i.e., use >1 tobacco product) [6,7] and rates of multi-product use are increasing [8]. Nationwide, 63% of past 30-day e-cigarette users in the 2014 National Youth Tobacco Survey (NYTS) also used at least one other tobacco product [6]. Moreover, certain combinations of poly-use are becoming increasingly common. In the 2011 NYTS, less than 1% of high school students reported dual use of combustibles with e-cigarettes or poly-use of combustibles with e-cigarettes and noncombustibles; by 2015, 6.6% reported dual-use combination and 2.6% reported poly-use combination [9].

Any use of nicotine products among youth is concerning because of the potential for nicotine to harm the developing brain [9,10,11] but dual and poly-use may be particularly problematic. For example, dual users of cigarettes and smokeless tobacco are exposed to higher levels of nicotine and have more difficulty quitting than users of either product on its own and dual users are likely to continue their dual use when followed over time, rather than quitting or switching to single product use [12,13]. High school students in the 2015 national Youth Risk Behavior Survey who were dual users of e-cigarettes and cigarettes used both products with greater intensity (i.e., more days in the past month) than cigarette-only or e-cigarette-only users [14], suggesting greater overall exposure to harmful constituents in cigarette smoke and e-cigarette aerosol. In that same study, dual users were also more likely than their single user counterparts to engage in other risky behaviors, such as using alcohol, marijuana and illicit drugs [14]. Together, these studies suggest that poly-use of tobacco products is associated with engagement in a cluster of risky behaviors and that there is a need for particular public health concern about dual and polytobacco product use among youth. Little is known about the processes that drive these patterns of use. Research on the factors associated with specific patterns of dual and poly-use among youth is needed to inform theoretical understanding of the uptake and trajectories of dual and poly-use. 

To date, studies suggest that use of e-cigarettes and cigarettes is not only related to demographic factors like sex, race and age but also to a variety of psychosocial factors. For example, in a 2014 California study, home use, friends’ use and positive attitudes toward e-cigarettes and cigarettes were associated with youths’ use of each product on its own [15]. Psychosocial factors are also associated with differences between multi-product users and single product users [16,17,18]. These factors include youths’ risk perceptions about tobacco-related health risks [16,18], perceived norms of peer non-cigarette tobacco use [16], believing that smoking is cool [16], thinking that smokers have more friends [16], having more positive beliefs about tobacco companies [17], having more positive beliefs about tobacco products [17] and being more exposed and more receptive to pro-tobacco marketing [18]. Few interventions targeting dual use exist [19] and future efforts to develop such interventions will need to take these types of psychosocial factors and tobacco-related beliefs into account.

The purpose of this study is to identify the demographic and psychosocial factors associated with four non-overlapping patterns of tobacco product use among middle and high school students: (1) e-cigarettes only; (2) e-cigarettes and cigarettes only; (3) e-cigarettes and other tobacco products (OTPs) only; and (4) e-cigarettes, cigarettes and OTPs. We focus only on the population of e-cigarette users because e-cigarettes are currently the most widely used nicotine product among youth [2]. Given the previously documented importance of psychosocial factors to single, dual and poly-use, we examine perceptions of health risk, addictiveness, acceptability and social benefits, along with receptivity to tobacco marketing and behavioral and demographic characteristics. Understanding these factors could provide useful information about what youth e-cigarette users find appealing about e-cigarette products, whether public health efforts for e-cigarettes would benefit from addressing concurrent use with tobacco products and recommendations for attitudes and beliefs to address with health communications.

## 2. Methods

### 2.1. Data

#### Florida Youth Tobacco Survey (FYTS 2014)

Data are from the annual 2014 Florida Youth Tobacco Survey (FYTS), a school-based, pencil-and-paper questionnaire given to Florida middle (grades 6–8) and high school (grades 9–12) students. FYTS is a cross-sectional, representative state sample based on a two-stage cluster probability sample design. Data were statistically weighted to represent state-level estimates using the study sampling frame from the U.S. Department of Education based on population totals for each combination of students’ grade, race and sex. The 2014 data contain 69,923 youth: 36,993 middle school students and 32,930 high school. Overall participation rates were 81% for middle school and 78% for high school. All subjects gave their informed consent for inclusion before they participated in the study. The study was conducted in accordance with the Declaration of Helsinki and the protocol was reviewed and exempted as ongoing public health surveillance by the Florida Department of Health Institutional Review Board.

### 2.2. Measures

#### 2.2.1. Current Use

Survey items measuring current use of tobacco and marijuana varied in response options with some items that asked participants to report “yes” or “no” and others that asked for number of days out of the past 30. For all tobacco products, current use was defined as using the product on at least 1 of the past 30 days.

Current use of bidis, kreteks, pipe tobacco, flavored cigarettes, flavored cigars, flavored smokeless, hookah, e-cigarettes and snus, was measured using the question, “During the past 30 days, have you (smoked or used)…?” with a yes or no response option. Marijuana use in a cigar was measured using the question “During the past 30 days, did you only smoke cigars that had marijuana in them?” Items used to measure current use of cigarettes, cigars and smokeless tobacco were asked individually with the question, “During the past 30 days, on how many days did you (smoke cigarettes; smoke cigars, cigarillos, or little cigars; use chewing tobacco, snuff, or dip)?” We defined OTP use as past 30-day use of bidis, kreteks, tobacco in a pipe, snus, chew, cigars, smokeless tobacco, marijuana used in a blunt, flavored cigars, flavored smokeless products, and/or flavored cigarettes. Respondents who did not use e-cigarettes were excluded from the study (*N* = 65,281). This left *N* = 4642 cases of current e-cigarette users for analysis.

Based on current e-cigarette users’ responses, we categorized them as belonging to one of four, mutually exclusive groups of product user types: (1) e-cigarettes only (EC); (2) e-cigarettes and cigarettes without OTP use (EC/C); (3) e-cigarettes and OTPs without cigarette use (EC/OTP); and (4) e-cigarettes, cigarettes and one or more OTPs (EC/C/OTP). For example, adolescents reporting use of e-cigarettes and hookah but not cigarettes would be in the EC/OTP group while adolescents who reported using e-cigarettes, cigarettes and hookah would be in the EC/C/OTP group. Respondents with missing data or inconsistent answers (*N* = 1687, 6.8%) on any tobacco use question were excluded from regression analyses. 

#### 2.2.2. Demographics

Self-reported age was categorized into three categories: 9–14, 15–17 and 18 or older. Respondents reported their sex as either female or male. Race/ethnicity was measured using two items asking respondents to report their race (American Indian or Alaska Native, Asian, black or African American, Native Hawaiian or Other Pacific Islander, or white) and whether they identify as Hispanic (Mexican, Puerto Rican, Cuban, or some other Hispanic or Latino identity not listed). The following race/ethnicity categories were used for analysis: “white, non-Hispanic”, “black, non-Hispanic”, “Hispanic”, and “other, non-Hispanic”. Non-Hispanic multiracial participants were included in the “other, non-Hispanic” race/ethnicity category, while participants of any race were included in the “Hispanic” race/ethnicity category.

#### 2.2.3. Tobacco Use Risk Factors

##### Environmental Characteristics

Exposure to tobacco products is an environmental factor known to be associated with e-cigarette and tobacco use [6,20,21] and was included in the model. Respondents were asked “Does anyone who lives in your home use any of the following products now? (Do not count yourself)” for cigarettes, cigars, chew, hookah and e-cigarettes. Indicators were created for each product type for youth responding “yes.” In addition to these indicators, we also included an indicator of exposure to cigarette smoke for youth reporting 1 or more days in the past week they had been in a room with someone smoking.

##### Psychosocial Characteristics

Given their relationship with e-cigarette and tobacco use in other studies [6,15,20,21], psychosocial characteristics included school performance, respondents’ perceived acceptability of smoking among adults, beliefs about the relative harm of e-cigarettes compared to cigarettes, receptivity to tobacco company marketing, whether the youth thought they would be smoking in 5 years, whether respondents felt the five tobacco products were easy to quit using and a scale for how positive respondents felt about the five products (cigarettes, cigars, chew, hookah and e-cigarettes). All items were asked of e-cigarette users.

For school performance, we asked youth to report what letter grade they most often received based on the typical U.S. descending scale of A, B, C, D, or F. Youth reporting that they received mostly As, Bs, or Cs were coded as having “good” grades, while youth reporting that they received mostly Ds or Fs were coded as having “poor” grades.

Perceived smoking acceptability was created using the question “Do you think your friends view cigarette smoking among adults as acceptable?” Responses were grouped into two categories of “Definitely Not” and “Probably Not” versus “Definitely Yes” and “Probably Yes”.

A measure of how risky respondents thought e-cigarettes were compared to cigarettes was created using the question “Compared to cigarette smoking, electronic cigarette (e-cigarette) smoking or ‘vaping’ is…” with youth reporting “Equally harmful” and “More harmful” = 0 and “Less harmful” = 1, such that a higher value for the item equates to thinking e-cigarettes are less harmful than cigarettes.

Receptivity to tobacco company marketing was measured by asking respondents if they “Would wear or use something with a tobacco brand on it (lighter, t-shirt, hat, sunglasses, etc.)?” Youth responding, “Definitely Yes” or “Probably Yes” were coded as receptive.

Youth were asked to report whether they thought they would be smoking cigarettes 5 years from now. Responses were grouped into two categories of “Definitely Not” and “Probably Not” versus “Definitely Yes” and “Probably Yes”.

Respondents were also asked “Do you think it would be easy to quit using the following products” for cigarettes, cigars, chew, hookah and e-cigarettes. Youth responding, “Definitely Yes” or “Probably Yes” were coded as thinking the product would be easy to quit and youth responding, “Definitely Not” or “Probably Not” were coded as not thinking the product would be easy to quit.

Finally, to assess how positively respondents felt about the five tobacco products (cigarettes, cigars, chew, hookah and e-cigarettes) a scale was created from responses to the four dichotomized questions for each product type. The questions were: “Do you think young people who use the following products have more friends?”; “Do you think using the following products makes young people look cool or fit in?”; “… helps people feel more comfortable at parties or in other situations?”; and “…help people relieve stress?” Responses to each item were dichotomized such that “Definitely Not” or “Probably Not” = 0 and “Definitely Yes” or “Probably Yes” = 1. The scale for each product ranges from 0 to 4, with 0 being disagreement with all statements about the product and 4 being agreement with all statements about the product. Cronbach’s alphas for the scales ranged from 0.64 to 0.69.

### 2.3. Analysis

Weighted estimates of the prevalence of the four product use categories were calculated. Weighted bivariate analyses were conducted to assess unadjusted differences in demographics, environmental factors and psychosocial characteristics for the four e-cigarette user types using chi-square tests. Regression analyses were limited to cases with complete data for all variables in the model, resulting in an analytic sample of *N* = 2756. Associations between e-cigarette user type and all characteristics were examined using weighted multinomial logistic regression, with exclusive e-cigarette users (the EC group) as the referent group. The model adjusted for covariates including age, race, gender and self-reported school performance and assessed for collinearity using variance inflation factor tests. All analyses were completed in 2017 using Stata version 14 (StataCorp LLC, College Station TX, USA) using the svy suite to account for sample clustering. Statistical significance is reported at *p* < 0.05.

## 3. Results

In Florida, 20.5% of high school students and 8.5% of middle school students currently use e-cigarettes [4]. The majority (81.2%) of e-cigarette-using youth in Florida also used tobacco products (Table 1). A total of 18.8% of current e-cigarette-using youth exclusively used e-cigarettes (EC group) and 3.3% exclusively used e-cigarettes and cigarettes (EC/C group). Almost 80% of youth e-cigarette users reported concurrent use of non-cigarette OTPs, 40.2% in the EC/C/OTP group and 37.8% in the EC/OTP group (Table 2). Youth in the EC/C/OTP group used an average of 4.4 OTPs, while youth in the EC/OTP group used an average of 2.4 OTPs. Table 2 also presents the weighted bivariate analyses assessing unadjusted differences in demographics, environmental factors and psychosocial characteristics for the four e-cigarette user types using chi-square tests.

We report results from multinomial logistic regression in Table 3. Compared with the EC group, living with someone who uses e-cigarettes was negatively associated with use of any tobacco products. Relative risk ratios (RRR) show that living with someone who uses hookah was significantly associated with a greater likelihood of EC/OTP use (RRR = 3.17, *p* < 0.0001) and EC/C/OTP use (RRR = 2.13, *p* < 0.001). Living with someone who uses cigars was significantly associated with a greater likelihood of EC/C use (RRR = 2.41, *p* < 0.05) and EC/C/OTP use (RRR = 2.15, *p* < 0.001). Relative to the EC group, having been in a room with a smoker in the past 30 days was significantly associated with a greater likelihood of being in the EC/OTP group (RRR = 1.66, *p* < 0.001) and EC/C/OTP group (RRR = 3.22, *p* < 0.0001). 

Compared with the EC group, none of the three multiple product user types showed significant differences in association with perceiving e-cigarettes as more harmful than cigarettes. Thinking that cigarettes would be easy to quit was associated with a greater likelihood of EC/OTP use (RRR = 2.51, *p* < 0.001) and EC/C/OTP use (RRR = 3.20, *p* < 0.0001) relative to exclusive EC use. Relative risk ratios indicate respondents who thought they will be smoking cigarettes in 5 years had a greater likelihood of being in the cigarette use groups (EC/C users (RRR = 4.04, *p* < 0.0001) and EC/C/OTP users (RRR = 7.82, *p* < 0.0001). Few RRRs for positive product attitudes showed statistical associations with the user types relative to the EC group and results were mixed. Relative to the EC group, tobacco company marketing receptivity was significantly associated with a greater likelihood of EC/C use (RRR = 2.00, *p* < 0.05), EC/OTP use (RRR = 2.11, *p* < 0.0001) and EC/C/OTP use (RRR = 2.09, *p* < 0.0001).

## 4. Discussion

Results showed that a large majority of e-cigarette-using youth in Florida also use tobacco products and that a significant proportion used more than four products on average. This suggests that any effort to prevent or address e-cigarette use among youth should be done with consideration for concurrent use with multiple tobacco products. In addition, nearly a fifth of e-cigarette using youth did not use tobacco products. This suggests that some youth are being exposed to nicotine who might not otherwise use tobacco products. This exposure is concerning due to the harms nicotine can pose to brain development during adolescence, the potential for youth to become nicotine dependent and the potential for increased susceptibility for using tobacco products, which contain many deadly carcinogens. 

We do not know the degree to which youth e-cigarette use is part of a temporal experimentation process or will escalate to sustained use of multiple nicotine products. However, even brief periods of concurrent use of multiple nicotine products pose health risks to children because multiple product use can be associated with exposure to higher levels of nicotine [12], that is harmful to adolescent brain development [11,21,9]. When followed over time, dual users are much more likely to continue to dual use than to quit or to switch to single product use [13], suggesting that they might experience greater difficulty quitting [12]. These risks underscore the need for public health efforts to prevent or reduce concurrent use of e-cigarettes with cigarettes or OTP among youth. Further research is needed to develop effective prevention and health communication strategies for addressing the concurrent use of e-cigarettes and multiple tobacco products among youth. 

Some psychosocial correlates had common associations across the four groups of user types. The consistently high percentages of agreement and association with the belief that e-cigarettes are less harmful than smoked cigarettes across the e-cigarette user types suggest that all e-cigarette-using youth might respond similarly to messages related to harm. Such messages could be cross-cutting and would not need to be targeted to a specific user type.

However, some findings suggest that there are differences in psychosocial and demographic characteristics across the four types of e-cigarette-using youth. Social exposure to tobacco use, both living with and being in the same room with a tobacco user, was associated with dual and poly use. This finding highlights the potential value of encouraging parents to stop using tobacco and to extend any home rules about tobacco use to include both cigarettes and non-cigarette OTPs.

These data showed believing that it is easy to quit smoking is positively associated with using e-cigarettes concurrently with OTPs relative to exclusive use of e-cigarettes, regardless of whether the youth smoked. Furthermore, thinking that oneself will be smoking cigarettes in 5 years was strongly associated with e-cigarette user types that concurrently smoke cigarettes. This suggests that addiction-related perceptions have a nuanced association with polytobacco product use. Although the general belief that quitting smoking is easy was associated with use of additional tobacco products beyond e-cigarettes, current cigarette smoking was associated with an understanding among youth that they would still be smoking in 5 years. Further research is needed to understand how these perceptions might be related to experimentation with multiple tobacco products and progression to nicotine dependence.

Finally, our results also showed that receptivity to tobacco company marketing was significantly associated with all of the multiple product user types relative to e-cigarette only users, similar to the results found in other research on multiple product use [18]. Receptivity can be successfully influenced by public health efforts. For example, the “truth” antismoking campaign, which focused on countering tobacco industry marketing tactics, reduced youth smoking prevalence [22]. Thus, similar denormalization efforts could be a promising approach for addressing youth polytobacco use among e-cigarette-using youth.

Results reported here benefit from the use of FYTS, a state-level representative dataset that captures relatively large numbers of current e-cigarette-using youth. However, there are several limitations that should be noted. The FYTS is cross-sectional and did not collect data on the use history of participants; therefore, we cannot assess use trajectories or potential gateway effects. The FYTS is also a school-based sample of youth in Florida and may not adequately represent youth not in school, especially those who have dropped out of enrollment. We were unable to control for some factors, such as socioeconomic status, that may be associated with e-cigarette and tobacco product use. 

## 5. Conclusions

Prevention efforts for e-cigarettes are novel and often do not specifically address use of multiple nicotine products. Understanding variation in psychosocial characteristics among types of e-cigarette users could provide useful information for developing effective prevention efforts for tobacco use among e-cigarette users. In addition to demographic factors, psychosocial factors may put some youth at greater risk for e-cigarette polytobacco use and increased exposure to nicotine and other harmful chemical constituents in tobacco products. Our results suggest that prevention efforts and health communications might benefit from addressing dual and polytobacco use patterns, and associated psychographic characteristics specifically. Although effective messages and strategies for e-cigarettes and dual or polytobacco use are yet to be developed and tested, our findings suggest that youth perceptions about ease of quitting cigarettes, intentions to continue smoking cigarettes, and receptivity to tobacco company marketing are promising areas for media campaign messaging aimed at reducing tobacco product use among youth. Results also showed that being in the same room with a smoker is associated with increased tobacco product use, suggesting potential importance of reducing exposure to tobacco use. Home rules about tobacco use and clean indoor air policies reduce youth cigarette smoking [11] but little is known about whether these factors similarly influence e-cigarette and polytobacco use. Further research may determine if expansion of such policies is effective for reducing youth e-cigarette and polytobacco use and through what pathways (e.g., changing social norms).

## Figures and Tables

**Table 1:** Current tobacco product use among youth current e-cigarette users in the Florida, Florida Youth Tobacco Survey, 2014.

Current E-Cigarette & Tobacco Product Use	Overall	
	*N*	Weighted %
		(95% CI)
EC use only	885	18.8%
EC/C use	186	3.3%
EC/OTP use	1537	37.8%
Mean number of OTPs used in EC/OTP group	2.4	(2.3, 2.5)
EC/C/OTP use	2034	40.2%
Mean number of OTPs used in EC/C/OTP group	4.4	(4.2, 4.5)

EC = e-cigarette. C = cigarette. OTP = other tobacco product.

**Table 2:** Characteristics among Types of Current E-cigarette Using Youth in Florida, Florida Youth Tobacco Survey, 2014.

Current Tobacco Product User Type	EC		EC/C		EC/OTP		EC/C/OTP		Chi-Square, *p*-Value
User Characteristics	*N*	Weighted %	*N*	Weighted %	*N*	Weighted %	*N*	Weighted %	
Lives with someone who uses:									
Cigarettes	375	38.5%	109	51.2%	635	42.7%	1180	62.1%	<0.001
Cigars	113	12.9%	35	19.0%	354	27.1%	769	30.0%	<0.001
Chew	88	7.2%	29	13.5%	317	17.6%	690	35.3%	<0.001
Hookah	74	9.1%	10	7.2%	386	27.8%	645	38.8%	
E-Cigarettes	335	37.3%	59	27.3%	558	38.9%	911	49.9%	<0.001
Days respondent was in the room with a smoker in the past week									
0 days	375	49.8%	44	26.3%	544	40.2%	307	19.5%	<0.001
1 or more days	470	50.3%	136	73.7%	876	59.8%	1552	80.5%	
Friends view smoking cigarettes among adults as acceptable									
Definitely no/Probably no	468	57.7%	75	38.5%	759	52.5%	607	33.0%	<0.001
Definitely yes/Probably yes	406	42.3%	110	61.5%	757	47.5%	1379	67.0%	
Compared with cigarette smoking, thinks e-cigarette use is									
Equally or more harmful	39	6.2%	15	9.2%	170	13.5%	284	21.5%	<0.001
Less harmful	692	93.8%	140	90.8%	1066	86.5%	1263	78.5%	
Believes it is “easy to quit using” the product (Definitely yes/Probably Yes)									
Cigarettes	116	10.6%	43	28.1%	325	21.2%	792	44.9%	<0.001
Cigars	242	29.0%	66	40.7%	552	37.0%	975	55.2%	<0.001
Chew	188	22.7%	60	39.0%	406	27.0%	858	49.9%	<0.001
Hookah	417	54.9%	90	62.1%	883	64.9%	1152	66.1%	<0.001
E-cigarettes	537	65.8%	99	61.3%	892	63.1%	1198	65.9%	
Will be smoking cigarettes in 5 years									
No	779	91.7%	106	65.5%	1266	86.1%	814	43.8%	<0.001
Yes	88	8.4%	76	34.5%	227	13.9%	1148	56.2%	
Positive product attitudes scale ^a^									
Cigarettes	1.8	(1.7, 1.9)	2.2	(2.0, 2.5)	1.9	(1.8, 1.9)	2.7	(2.6, 2.8)	<0.001
Cigars	1.6	(1.5, 1.7)	1.8	(1.5, 2.0)	1.8	(1.7, 1.9)	2.4	(2.3, 2.5)	<0.001
Chew	1.4	(1.3, 1.5)	1.7	(1.5, 2.0)	1.7	(1.6, 1.8)	2.3	(2.2, 2.3)	<0.001
Hookah	2.2	(2.0, 2.3)	2.1	(1.9, 2.4)	2.5	(2.4, 2.6)	2.8	(2.7, 2.8)	<0.001
E-cigarettes	2.2	(2.0, 2.3)	2.3	(2.1, 2.6)	2.3	(2.3, 2.4)	2.7	(2.6, 2.8)	<0.001
Would wear or use something with a tobacco brand on it (lighter, t-shirt, hat, sunglasses, etc.)									
Definitely no/Probably no	641	79.2%	97	57.0%	841	61.2%	704	39.8%	<0.001
Definitely yes/Probably yes	195	20.8%	81	43.0%	567	38.8%	1150	60.2%	
Age									
12–14	384	35.8%	50	20.4%	458	23.7%	540	21.3%	<0.001
15–17	442	56.8%	108	60.5%	903	62.8%	1107	57.5%	
18 or older	57	7.4%	28	19.1%	172	13.6%	379	21.2%	
Gender									
Female	421	43.1%	116	57.0%	670	39.7%	826	41.6%	<0.05
Male	454	56.9%	68	43.0%	842	60.3%	1156	58.4%	
Race/ethnicity									
White, non-Hispanic	569	55.2%	148	70.7%	900	48.7%	1191	49.1%	<0.001
Black, non-Hispanic	65	9.9%	7	3.3%	116	9.5%	156	9.9%	
Hispanic	169	25.9%	20	19.0%	395	35.2%	479	31.5%	
Other, non-Hispanic (includes Multi, non-Hispanic)	77	9.0%	9	7.0%	113	6.6%	184	9.6%	
Grades in school									
Mostly A/B/Cs	770	95.8%	143	88.2%	1287	92.1%	1393	77.7%	<0.001
Mostly D/Fs	45	4.2%	24	11.8%	120	7.9%	405	22.3%	

EC = e-cigarette. C = cigarette. OTP = other tobacco product. ^a^ The positive attitudes measures are on a scale of 0 to 4 with 0 = disagreement with all four statements about the product and 4 = agreement with all four statements about the products. The statements are as follows “Do you think young people who use the following products have more friends”, “Do you think using the following products makes young people look cool or fit in”, “Do you think using the following products helps people feel more comfortable at parties or in other situations”, and “Do you think using the following products helps people relieve stress?”.

**Table 3:** Current Tobacco Product Use among Youth Current Smokers in Florida, Florida Youth Tobacco Survey, 2014.

Current Tobacco Product User Type	EC/C		EC/OTP		EC/C/OTP	
Characteristic	RRR	[95% CI]	RRR	[95% CI]	RRR	[95% CI]
Live with someone who uses:						
Cigarettes	1.273	[0.664, 2.439]	0.967	[0.678, 1.379]	1.057	[0.724, 1.542]
Cigars	2.410 *	[1.181, 4.919]	1.422	[0.904, 2.237]	2.146 **	[1.343, 3.429]
Chew	1.268	[0.523, 3.075]	1.313	[0.817, 2.112]	1.459	[0.873, 2.438]
Hookah	0.601	[0.191, 1.891]	3.165 ***	[1.951, 5.133]	2.125 **	[1.238, 3.647]
E-cigarettes	0.386 **	[0.188, 0.791]	0.573 **	[0.399, 0.824]	0.515 **	[0.344, 0.770]
Days respondent was in the room with a smoker in the past week						
0 days	Ref.		Ref.		Ref.	
1 or more days	1.711	[0.872, 3.358]	1.660 **	[1.193, 2.311]	3.219 ***	[2.210, 4.689]
Friends view smoking cigarettes among adults as acceptable						
Definitely no/Probably no	Ref.		Ref.		Ref.	
Definitely yes/Probably yes	1.797	[0.957, 3.375]	1.147	[0.851, 1.546]	1.448 *	[1.037, 2.022]
Compared with cigarette smoking e-cigarette use is						
Equally or more harmful	Ref.		Ref.		Ref.	
Less harmful	1.136	[0.402, 3.206]	0.728	[0.382, 1.385]	0.802	[0.418, 1.536]
Believes it is “easy to quit using”						
Cigarettes	2.254	[0.791, 6.425]	2.509 **	[1.389, 4.533]	3.203 ***	[1.766, 5.809]
Cigars	0.559	[0.251, 1.245]	0.98	[0.659, 1.456]	1.073	[0.675, 1.704]
Chew	2.025	[0.878, 4.673]	0.594 *	[0.370, 0.955]	1.372	[0.811, 2.321]
Hookah	1.746	[0.642, 4.748]	1.880 **	[1.271, 2.779]	1.53	[0.958, 2.443]
E-cigarettes	0.529	[0.204, 1.369]	0.557 **	[0.384, 0.806]	0.498 **	[0.322, 0.769]
Will be smoking cigarettes in 5 years						
No	Ref.		Ref.		Ref.	
Yes	4.042 ***	[2.011, 8.126]	1.518	[0.871, 2.645]	7.818 ***	[4.716, 12.96]
Positive product attitudes scale ^a^						
Cigarettes	1.49	[0.975, 2.277]	0.629 ***	[0.503, 0.788]	1.098	[0.858, 1.405]
Cigars	0.847	[0.599, 1.198]	1.169	[0.967, 1.414]	1.078	[0.873, 1.331]
Chew	1.119	[0.828, 1.513]	1.181	[0.996, 1.399]	0.861	[0.714, 1.039]
Hookah	0.694 **	[0.528, 0.912]	1.189 *	[1.008, 1.403]	1.142	[0.935, 1.394]
E-cigarettes	0.994	[0.744, 1.328]	0.993	[0.839, 1.175]	0.904	[0.744, 1.098]
Would wear or use something with a tobacco brand on it (lighter, t-shirt, hat, sunglasses, etc.)						
No	Ref.		Ref.		Ref.	
Yes	1.995 *	[1.064, 3.742]	2.110 ***	[1.500, 2.968]	2.086 ***	[1.446, 3.010]
Age						
12–14	Ref.		Ref.		Ref.	
15–17	2.139 *	[1.066, 4.291]	1.904 ***	[1.361, 2.662]	2.389 ***	[1.623, 3.517]
18 or older	4.930 **	[1.577, 15.41]	2.664 **	[1.473, 4.816]	5.950 ***	[3.110, 11.38]
Gender						
Female	Ref.		Ref.		Ref.	
Male	0.487 *	[0.260, 0.910]	1.197	[0.882, 1.624]	0.941	[0.676, 1.311]
Race/ethnicity						
White, non-Hispanic	Ref.		Ref.		Ref.	
Black, non-Hispanic	0.635	[0.196, 2.061]	0.927	[0.517, 1.663]	1.117	[0.572, 2.183]
Hispanic	0.588	[0.202, 1.713]	1.936 **	[1.285, 2.915]	1.088	[0.681, 1.739]
Other, non-Hispanic (includes Multi, non-Hispanic)	0.52	[0.154, 1.751]	0.848	[0.510, 1.409]	0.768	[0.430, 1.372]
Grades in school						
Mostly A/B/Cs	Ref.		Ref.		Ref.	
Mostly D/Fs	2.019	[0.831, 4.902]	0.912	[0.498, 1.673]	3.083 ***	[1.715, 5.545]

* *p* < 0.05, ** *p* < 0.01, *** *p* < 0.001. EC = e-cigarette. C = cigarette. OTP = other tobacco product. ^a^ The positive attitudes measures are on a scale of 0 to 4 with 0 = disagreement with all four statements about the product and 4 = agreement with all four statements about the products. The statements are as follows “Do you think young people who use the following products have more friends”, “Do you think using the following products makes young people look cool or fit in”, “Do you think using the following products helps people feel more comfortable at parties or in other situations”, and “Do you think using the following products helps people relieve stress?”.
